# Overturning Roe v. Wade and pre-medical students’ views and medical school choices

**DOI:** 10.1080/10872981.2025.2585634

**Published:** 2025-11-15

**Authors:** Emma J. Hastings, M. Courtney Hughes, Lei Hua

**Affiliations:** a Department of Health Sciences, Northern Illinois University, DeKalb, IL, USA; b Department of Public Health, Northern Illinois University, DeKalb, IL, USA; c Department of Statistics and Actuarial Science, Northern Illinois University, DeKalb, IL, USA

**Keywords:** Abortion policy, medical school, reproductive rights, healthcare workforce, Roe v. Wade

## Abstract

The overturning of Roe v. Wade in Dobbs v. Jackson Women’s Health Organization (2022) has led to varying abortion laws across states in the U.S., potentially influencing medical education choices. While some research has examined abortion policy changes and medical residencies and fellowship decisions, none has investigated abortion policy and medical school decisions. This study aims to determine whether pre-medical students’ personal views on abortion influence their willingness to attend medical schools in states where abortion is illegal at all stages EXCEPT to save the life of the mother. A cross-sectional survey was distributed to pre-medical organizations at the two largest four-year institutions in each U.S. state and the District of Columbia, except Wyoming, which has only one four-year institution. The survey collected demographic data, political affiliation, intended medical specialty, and personal stance on abortion. Participants indicated their willingness to attend medical school in states with different types of abortion policies. There were 182 completed surveys from participants in 20 different states. Analysis showed that students who believed abortion is acceptable and should be legal at all stages were significantly less willing to attend medical school in states where abortion is illegal EXCEPT to save the life of the mother. Abortion policy may influence medical school decisions among pre-medical students, which may have long-term implications for physician distribution, particularly in states with restrictive abortion laws. Future research should explore how these trends impact healthcare workforce shortages and access to reproductive care.

## Introduction

Abortion, the termination of pregnancy through medical or procedural means, has been a contentious issue for decades [1]. The 1973 Roe v. Wade decision established abortion rights as a constitutional precedent but was overturned in Dobbs v. Jackson Women’s Health Organization (2022). Recent data shows a decline in residency applications in states with abortion bans [2]. In specializations such as family medicine and emergency medicine, limitations on abortion training could lead to lapses in patient care [3,4]. Similarly, for graduating residents, there has been a shift away from choosing to practice in states with abortion restrictions, particularly in fields like obstetrics and gynecology [5], which is heavily influenced by reproductive healthcare policies [6]. While research exists on abortion policy changes and the impact on medical residencies and fellowships [2,4], little is known about how abortion policies affect pre-medical students' medical school choices. This study examines whether students avoid states with the most restrictive abortion ban, when abortion is illegal at all stages EXCEPT to save the life of the mother.

## Materials and methods

This study employed a cross-sectional survey design to examine the relationship between personal beliefs on abortion and pre-medical students' willingness to attend medical school in states with varying abortion laws. An online survey was distributed to pre-medical organizations at the two largest four-year colleges in each U.S. state and the District of Columbia, except for Wyoming, which has only one four-year college, so it was distributed to one. Respondents were entered into a random drawing for a $50 prize. The survey included demographic questions, political affiliation, planned medical specialty, and personal stance on abortion. Participants were asked about their willingness to attend medical school in states with different abortion laws, which were categorized based on policies at the time of data collection. Eight states only allowed abortion to preserve the life of the mother: Alabama, Arkansas, Kentucky, Louisiana, Oklahoma, South Dakota, Tennessee, and Texas. The Northern Illinois University Institutional Review Board determined this study to be exempt from review in accordance with federal regulation criteria.

A proportional odds model and model diagnostics were used to assess students’ willingness to go to medical school in a state where abortion is illegal at all stages of the pregnancy EXCEPT to save the life of the mother. The feedback of ‘No,’ ‘Unsure,’ and ‘Yes’ were treated as ordinal response variables, and explanatory variables, including positions on abortion policies, political affiliations, and gender were attempted.

## Results

There were 182 respondents from participants in 20 different states (Table 1). We found that willingness to attend medical school in a state increases as abortion access becomes less restrictive, with the highest support (62.4%) when abortion is legal at all stages. Conversely, the most restrictive policy (abortion illegal EXCEPT to save the mother's life) has the lowest willingness (30.3%) and highest unwillingness (43.0%), with the remaining unsure (26.7%). We found that positions on abortion and political affiliations were significantly dependent, and the gender was not significant at a significance level of 5%. So, we chose to use the positions on abortion as the explanatory variable.

**Table 1.** Characteristics of the sample population.

Characteristic		Total (% of sample)
Race/Ethnicity		
	White	100 (59.9)
	Black or African American	14 (8.4)
	Asian	43 (25.7)
	American Indian or Alaska Native	3 (1.8)
	Native Hawaiian or Other Pacific Islander	2 (1.2)
	Hispanic or Latinx	21 (12.6)
	Middle Eastern or North African	6 (3.6)
Sex		
	Male	22 (13.2)
	Female	142 (85.0)
Relationship		
	Single	109 (65.3)
	Married	1 (0.6)
	Living with a partner	7 (4.2)
	In a committed romantic relationship	50 (29.9)
Political affiliation		
	Conservative	7 (4.2)
	Conservative leaning	20 (12.0)
	Moderate	35 (21.1)
	Liberal leaning	44 (26.5)
	Liberal	60 (36.1)
Planned specialty		
	Emergency medicine	21 (2.8)
	Family medicine	17 (10.4)
	Internal medicine	9 (5.5)
	OB/GYN	28 (17.1)
	Other	52 (31.7)
	Unsure	37 (22.6)
Sexually active in the past year		
	Yes	86 (55.5)
	No	72 (43.1)
Intends to go to medical school		
	Yes	137 (82.0)
	No	7 (4.2)
	Unsure	23 (13.8)
Year in college		
	Freshman	49 (29.3)
	Sophomore	41 (24.6)
	Junior	38 (22.8)
	Senior	30 (18.0)
	Gap Year	6 (3.6)
In what state do you attend college?		
	Arkansas	8 (4.8)
	California	2 (1.2)
	Colorado	1 (0.6)
	Hawaii	12 (7.2)
	Illinois	27 (16.3)
	Indiana	1 (0.6)
	Iowa	5 (3.0)
	Kansas	2 (1.2)
	Maine	1 (0.6)
	Massachusetts	7 (4.2)
	Michigan	2 (1.2)
	Missouri	1 (0.6)
	Nevada	2 (1.2)
	New York	1 (0.6)
	Oklahoma	22 (13.3)
	Oregon	14 (8.4)
	Texas	2 (1.2)
	Vermont	41 (24.7)
	Virginia	1 (0.6)
	Wisconsin	13 (7.8)

The following are the main results (*p* < 0.05):

1. Positions on abortion significantly affect willingness to go to medical schools in a state in which abortion is illegal at all stages EXCEPT to save the life of the mother (*p*-value: $3.1\times 10^{-5}$).
2. A post-hoc test using Bonferroni correction suggests that those respondents who believe having an abortion is acceptable and should be legal had significantly less willingness than any of the students who had other positions on abortion in responding to the question on willingness to go to medical schools in a state in which abortion is illegal at all stages EXCEPT to save the life of the mother.

## Discussion

Our study finds that pre-medical students who believe abortion should be legal at all stages were less likely to consider medical schools in states with strict abortion bans. Additionally, pre-medical students' willingness to attend medical schools in states where abortion is illegal in all cases EXCEPT to save the life of the mother is influenced by their personal views on abortion. These findings highlight the potential impact of abortion policies on future medical professionals' decisions and could have long-term implications for healthcare workforce distribution.

A strength of this study included its diverse sample across race and ethnicity, political affiliation, state, and planned medical specialty, enhancing the generalizability of our findings. A limitation included an overrepresentation of female and liberal-leaning respondents, which may affect the applicability to male or conservative populations. Additionally, students who chose to participate may be more interested in abortion policy or have stronger views on the topic than those who didn’t participate. Future research into abortion policies and how they shape pre-medical students’ perspectives and medical school decisions is vital for understanding potential effects on the future healthcare workforce.
